# Functional and Morphological Differences of Muscle Mitochondria in Chronic Fatigue Syndrome and Post-COVID Syndrome

**DOI:** 10.3390/ijms25031675

**Published:** 2024-01-30

**Authors:** Daniel Alexander Bizjak, Birgit Ohmayer, Jasmine Leonike Buhl, Elisabeth Marion Schneider, Paul Walther, Enrico Calzia, Achim Jerg, Lynn Matits, Jürgen Michael Steinacker

**Affiliations:** 1 Division of Sports and Rehabilitation Medicine, University Hospital Ulm, 89075 Ulm, Germanyjasmine-leonike.buhl@uniklinik-ulm.de (J.L.B.); achim.jerg@uniklinik-ulm.de (A.J.); lynn.matits@uni-ulm.de (L.M.); juergen.steinacker@uni-ulm.de (J.M.S.); 2 Clinic of Anaesthesiology and Intensive Care Medicine, University Hospital Ulm, 89081 Ulm, Germany; marion.schneider@uniklinik-ulm.de; 3 Central Facility for Electron Microscopy, Ulm University, 89081 Ulm, Germany; paul.walther@uni-ulm.de; 4 Institute for Anaesthesiologic Pathophysiology and Process Engineering, Ulm University, 89081 Ulm, Germany; enrico.calzia@uni-ulm.de; 5 Clinical & Biological Psychology, Institute of Psychology and Education, Ulm University, 89081 Ulm, Germany

**Keywords:** PASC, inflammation, chronic fatigue, mitochondrial dysfunction, oxidative phosphorylation, mitochondrial morphology

## Abstract

Patients suffering from chronic fatigue syndrome (CFS) or post-COVID syndrome (PCS) exhibit a reduced physiological performance capability. Impaired mitochondrial function and morphology may play a pivotal role. Thus, we aimed to measure the muscle mitochondrial oxidative phosphorylation (OXPHOS) capacity and assess mitochondrial morphology in CFS and PCS patients in comparison to healthy controls (HCs). Mitochondrial OXPHOS capacity was measured in permeabilized muscle fibers using high-resolution respirometry. Mitochondrial morphology (subsarcolemmal/intermyofibrillar mitochondrial form/cristae/diameter/circumference/area) and content (number and proportion/cell) were assessed via electron microscopy. Analyses included differences in OXPHOS between HC, CFS, and PCS, whereas comparisons in morphology/content were made for CFS vs. PCS. OXPHOS capacity of complex I, which was reduced in PCS compared to HC. While the subsarcolemmal area, volume/cell, diameter, and perimeter were higher in PCS vs. CFS, no difference was observed for these variables in intermyofibrillar mitochondria. Both the intermyofibrillar and subsarcolemmal cristae integrity was higher in PCS compared to CFS. Both CFS and PCS exhibit increased fatigue and impaired mitochondrial function, but the progressed pathological morphological changes in CFS suggest structural changes due to prolonged inactivity or unknown molecular causes. Instead, the significantly lower complex I activity in PCS suggests probably direct virus-induced alterations.

## 1. Introduction

Mitochondria are the main power system of the human body. By producing adenosine triphosphate (ATP) in the electron transport chain and using energy-related substrates synthesized in the Krebs cycle, they constantly contribute to normal physiological performance and energy supply [1]. Conversely, if mitochondrial dysfunction is apparent, the development of chronic physiological performance impairment may be a negative consequence [1,2]. One prominent example for this impairment shows itself with the symptoms of fatigue and activity decrease, and patients suffering from Myalgic Encephalomyelitis/chronic fatigue syndrome (ME/CFS) or post-COVID syndrome exhibit a reduced physiological performance capability, with detrimental effects on quality of life and exercise capacity [3,4]. The multifactorial symptom origins make a single disease cause and progression unlikely, but impaired mitochondrial function and morphology may play a pivotal role [5].

ME/CFS is a chronic, mostly post-viral, disease with a very broad clinical picture. Worldwide, the number of sufferers is estimated to be as high as 17–24 million [6,7]. The disease predominately develops during adolescence and between the ages of 30 and 40 years but can occur at any age. A systematic review and meta-analysis by Lim et al. showed that women are 1.5- to 2.0-fold more affected than their male counterparts [7]. In most patients, ME/CFS is preceded by infection with viral agents, such as Epstein–Barr virus (EBV), other herpes viruses, influenza viruses, or enteroviruses. In addition, infections with bacteria or fungi, due to surgery, as well as psychological states of emergency are described as triggering causes [8].

International studies show that at least 10–20% of all COVID-19-infected persons still suffer from long-term consequences of the disease six months after the initial infection [9,10]. These long-term sequelae are also referred to as post-COVID syndrome (PCS). PCS is defined here as all complaints that still exist three months after the acute infection or that have newly occurred within this period and can be understood as a consequence of COVID-19 disease, as defined by the National Institute for Care and Excellence (NICE) [11]. Also included here are exacerbations of preexisting underlying diseases. The most common complaints of patients include chronic fatigue (37.2%), cognitive impairment such as impaired concentration and memory (31.3%), respiratory problems (30.2%), taste or smell disorders (23.6%), and depression (21.1%). The majority of PCS affected individuals’ experience of the relief of symptoms, and, in some cases, even complete remission, after some time has passed since the onset of the disease [12].

It is noteworthy that the symptoms typical for PCS coincide to a large extent with the symptom complex of ME/CFS. A study by Kedor et al. [13] found that half of the included post-COVID-19 patients met the diagnostic criteria for ME/CFS six months after infection. Other predictions [14] calculate incidences of 20–50% of all COVID-19-infected individuals with subsequent ME/CFS symptoms. This association suggests that the etiology of the diseases may also be similar. Some studies suggest that structural changes in musculoskeletal mitochondria may also occur in ME/CFS [15,16,17] and PCS patients [18]. However, findings from ultrastructural analysis showed a relatively normal mitochondrial structure in ME/CFS [19], and no alterations in normal mitochondrial function was found in CFS myotubes or PBMCs, but these results are based on relatively small sample sizes [20]. Nevertheless, the impaired mitochondrial bioenergetics of ME/CFS may begin upstream of the mitochondrial respiratory chain, as impaired mitochondrial energy metabolism may also play a role [20]. Thus, in a number of studies, in peripheral blood mononuclear cells (leukocytes and monocytes) and in skeletal muscle, decreased ATP production was found; the decreased ATP levels indicate a metabolic defect (e.g., abnormal lactate response or defect of oxidative metabolism with accelerated glycolysis) and decreased electron transport chain activity [17,21,22,23]. However, another study group examining a similar cohort published contradictory results and even demonstrated increased ATP production in ME/CFS patients, albeit by non-mitochondrial sources [24].

Hence, to further elucidate these complex mechanistic fatigue origins in ME/CFS and PCS as well as additional incongruent mitochondrial-based disease contribution, we took muscle biopsy samples from patients suffering from CFS and PCS as well as from healthy controls, and we examined the physiological and cellular differences between these groups with a special focus on the mitochondrial oxidative function capacity and potential structural abnormalities.

## 2. Results

Exercise Testing and anthropometry

**Figure 1:**
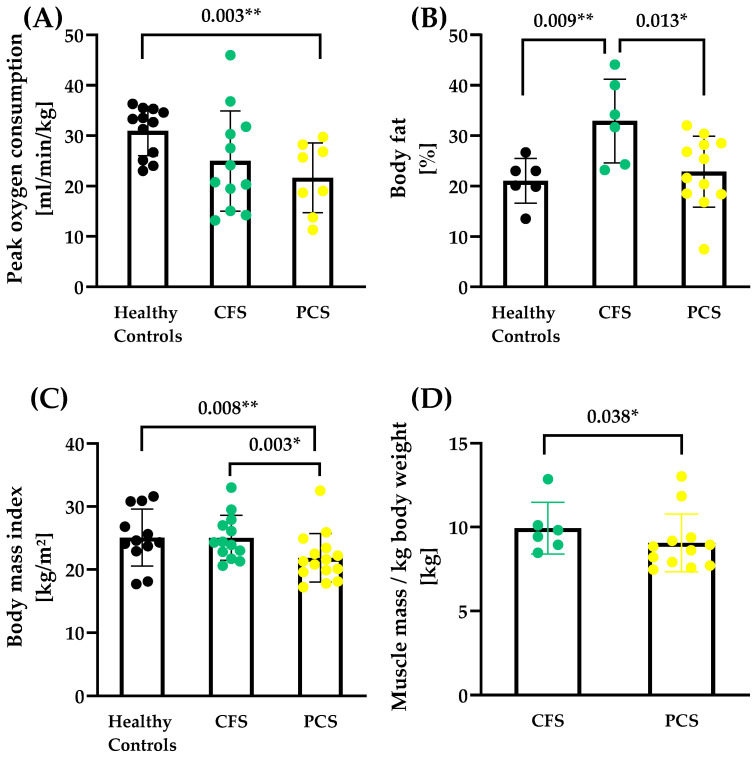
Performance capacity and body composition of CFS, PCS, and healthy controls (HCs). (**A**) Peak oxygen consumption (PCS N = 8, CFS N = 12; HC N = 11), (**B**) body fat (PCS N = 12, CFS N = 6; HC N = 6), (**C**) body mass index (PCS N = 12, CFS N = 6; HC N = 6) and (**D**) total muscle mass/kg body weight (PCS N = 12, CFS N = 6) are shown. Muscle mass data for healthy controls were not available as the measurement technique of the reference study was different. Significance set at *p*-value of * *p* ≤ 0.050, ** *p* ≤ 0.010. Results based on robust one-way ANOVA comparison between healthy controls, CFS and PCS. Post hoc tests were applied based on robust linear regression models with *p*-value adjustment according to Holm.

Even after testing for sex effects, healthy controls (HCs) showed a higher aerobic capacity reflected by VO_2peak_ (30.94 ± 4.87 mL/min/kg) compared to PCS patients (21.65 ± 6.93 mL/min/kg, *p* = 0.003) (Figure 1A). Three patients of CFS and seven patients of PCS could not perform the CPET due to acute high fatigue or danger of heavy post-exertional malaise. There was a significant difference in body fat (F (2, 11.2) = 4.53, HC vs. CFS *p* = 0.009; CFS vs. PCS *p* = 0.013; Figure 1B) and body mass index (BMI, F (2, 24.8) = 3.91, HC vs. PCS *p* = 0.008; CFS vs. PCS *p* = 0.003; Figure 1C). Also, a significant difference in muscle mass/kg was observed between CFS (9.94 ± 1.54 kg) and PCS (9.06 ± 1.71 kg) (Δ_Means trimmed_= 0.98, T_y_ = 1.97, *p* = 0.038, Figure 1D). All patients’ characteristics and their anthropometric data are shown in Table 1.

OXPHOS capacity

**Figure 2:**
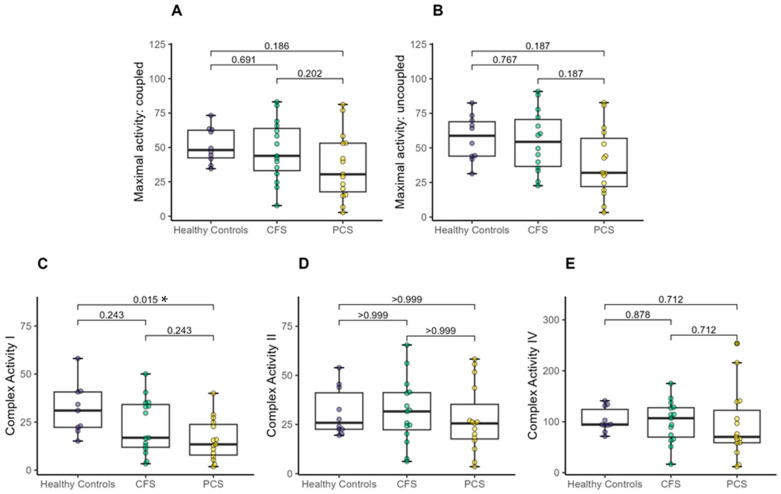
Mitochondrial oxidative phosphorylation (OXPHOS) capacity between CFS, PCS and healthy controls (PCS (13f/2m); CFS (10f/5m); healthy controls (4f/6m). (**A**) Maximal activity: coupled, (**B**) Maximal activity: uncoupled, (**C**) Complex I activity, (**D**) Complex II activity and (**E**) Complex IV activity. Significance set at *p*-value of * *p* ≤ 0.050. Results based on robust one-way ANOVAs comparison between healthy controls, CFS and PCS. Post hoc tests were applied based on robust linear regression models with *p*-value adjustment according to Holm.

Oxidative phosphorylation capacity via high-resolution respirometry revealed lower complex I activity of PCS compared to age-matched healthy controls. There was no difference in maximal coupled or uncoupled activity, or in complex II and complex IV activity between the groups (Figure 2A–E).

However, a one-way MANOVA showed no significant difference between healthy controls, patients with CFS, and patients with PCS (X^2^ (10) = 16.71, *p* < 0.081; Wilk’s Λ = 0.592).

Electron microscopy analysis

**Figure 3:**
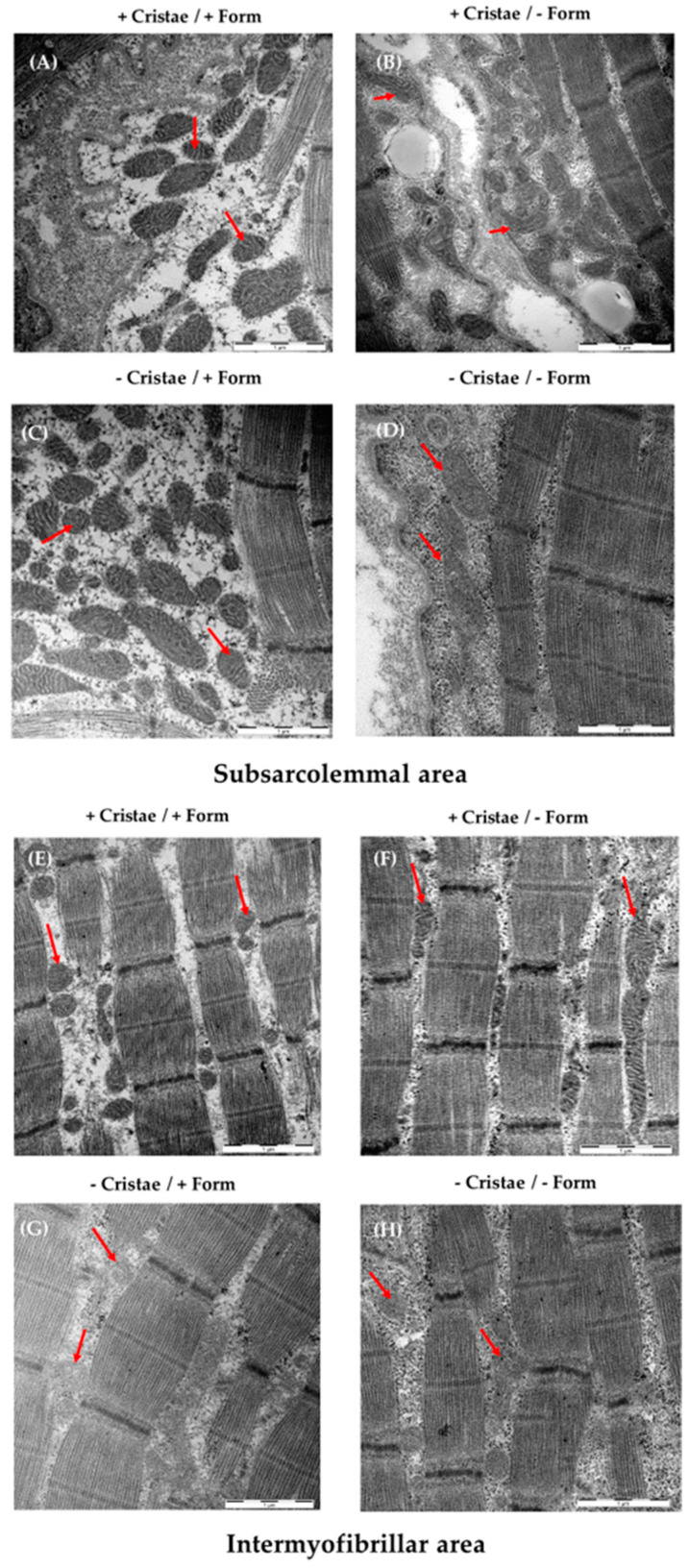
Examples of mitochondrial morphology of the M. quadriceps femoris (subsarcolemmal/intermyofibrillar mitochondrial form/cristae/diameter/circumference/area) and content (number and proportion/cell) assessed by electron microscopy (EM) and analyzed by ImageJ. Shown are EM images of muscle fibers exhibiting high cristae and uniform shape (+/+, (**A**,**E**)), high cristae and irregular shape (+/−, (**B**,**F**)), few cristae and regular shape (−/+, (**C**,**G**)), and few cristae and irregular shape (−/−, (**D**,**H**)). Representative mitochondria for each subtype are marked with red arrows. The scale bar at the right corner represents 1 µM.

Representative electron microscopy images that show the gradual difference between the inconspicuous mitochondrial shape and the conspicuous one, separated by subsarcolemmal and intermyofibrillar mitochondria, are presented in Figure 3. The cristae, form, diameter, circumference as well as the subsarcolemmal and intermyofibrillar area could be reliably measured and analyzed. Distinctive mitochondrial subpopulations of muscle with regard to cristae score or uniform shape/form could be determined. While the sarcomere structure is nearly intact and uniform in all muscle samples, mitochondria exhibited several different morphological features (shape and cristae) that assume a heterogeneous mitochondria population contributing to muscle impairment.

A and B are mainly found in in the subsarcolemmal mitochondria of PCS, whereas C and D are typically observed in ME/CFS.

**Figure 4:**
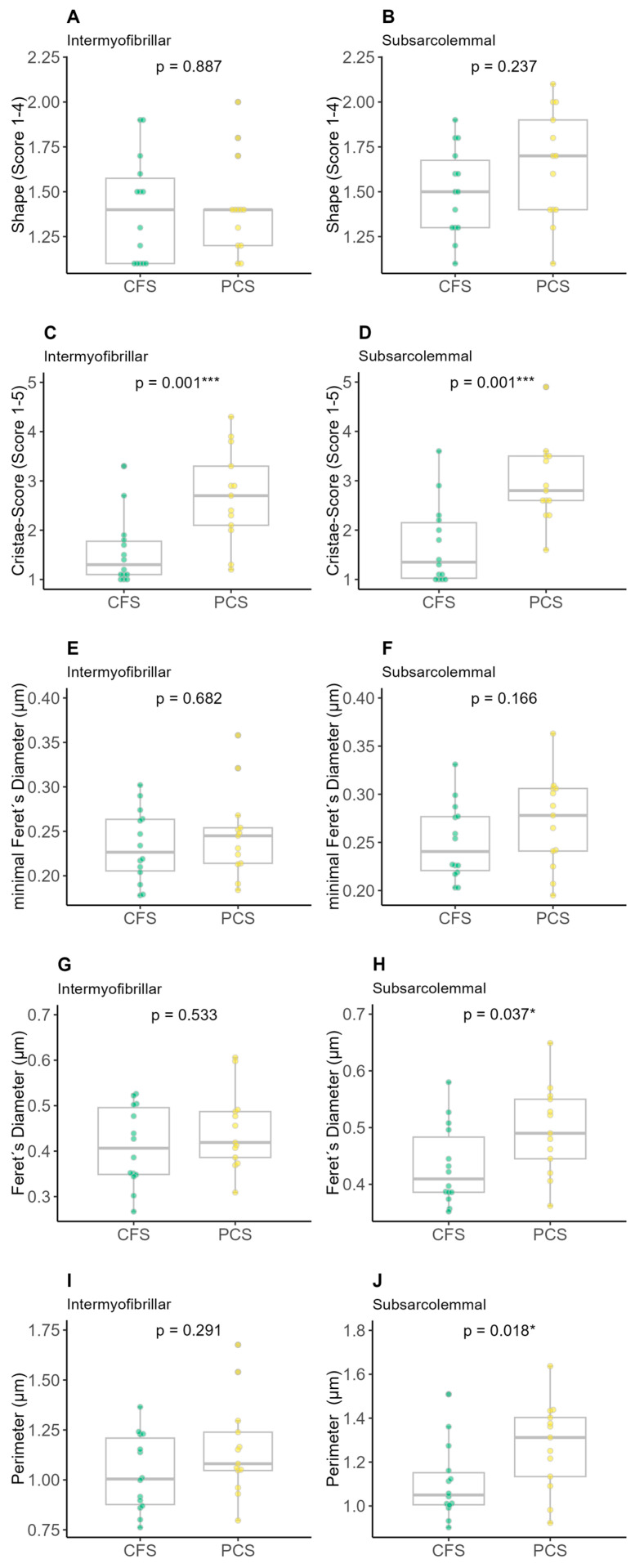
Results of electron microscopy (EM) analysis of chronic fatigue syndrome (CFS) and post-COVID syndrome (PCS) patients’ skeletal mitochondrial shape (PCS (12f/2m); CFS (9f/5m); (**A**) intermyofibrillar shape, (**B**) subsarcolemmal shape, (**C**) intermyofibrillar cristae score, (**D**) subsarcolemmal cristae score, (**E**) intermyofibrillar minimal Feret’s diameter, (**F**) subsarcolemmal minimal Feret’s diameter, (**G**) intermyofibrillar Feret’s diameter, (**H**) subsarcolemmal Feret’s diameter, (**I**) intermyofibrillar perimeter, (**J**) subsarcolemmal perimeter; significance set at *p*-value of * *p* ≤ 0.050, *** *p* ≤ 0.001. Differences in EM parameters were assessed using unpaired two-sample trimmed mean test (20%) with 10,000-fold bootstrap.

There was no difference between intermyofibrillar or subsarcolemmal shape score (Figure 4A,B) and minimal Feret’s diameter (Figure 4E,F) as well as in the intermyofibrillar Feret’s diameter or the perimeter between CFS and PCS. Significant differences were detected with a higher intermyofibrillar (2.97 ± 0.82 vs. 1.69 ± 0.82; *p* = 0.001) and subsarcolemmal (2.70 ± 0.96 vs. 1.56 ± 0.69; *p* = 0.001) cristae score for PCS compared to CFS (Figure 4C,D) as well as higher subsarcolemmal values of PCS regarding Feret’s diameter (0.50 ± 0.08 vs. 0.43 ± 0.07 µm; *p* = 0.037, Figure 4H) and perimeter (1.27 ± 0.20 vs. 1.11 ± 0.17 µm; *p* = 0.018, Figure 4J).

**Figure 5:**
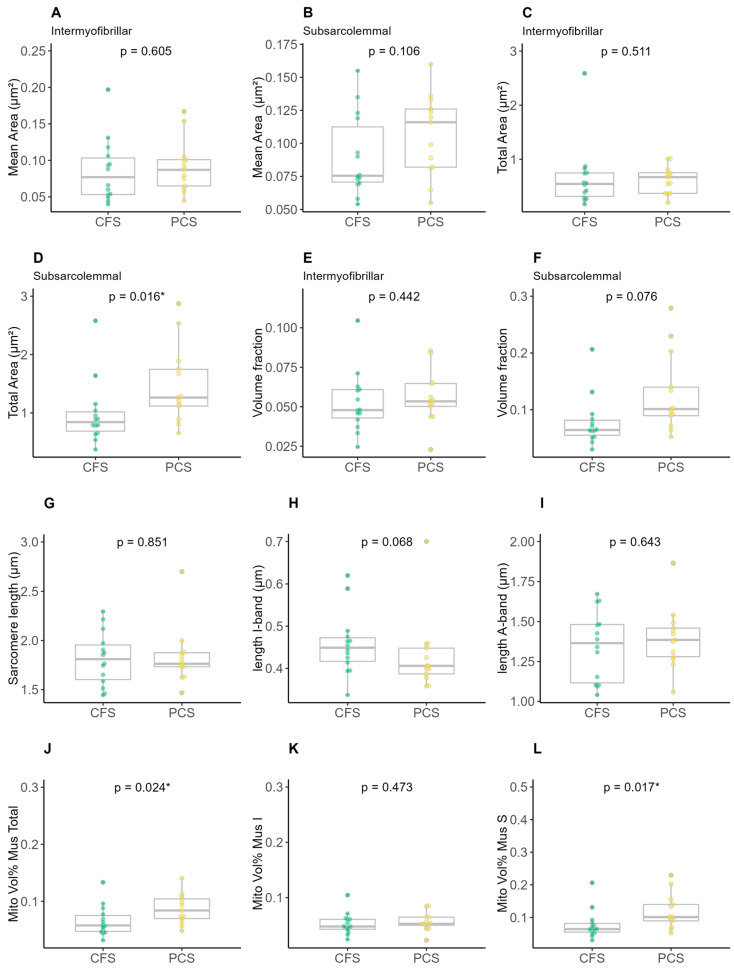
Results of electron microscopy analysis of chronic fatigue syndrome (CFS) and post-COVID syndrome (PCS) patients’ skeletal mitochondrial morphology (PCS (12f/2m); CFS (9f/5m); (**A**) intermyofibrillar mean area, (**B**) subsarcolemmal mean area, (**C**) intermyofibrillar total area, (**D**) subsarcolemmal total area, (**E**) intermyofibrillar volume fraction, (**F**) subsarcolemmal volume fraction, (**G**) sarcomere length, (**H**) length I-band, (**I**) length A-band, (**J**) total mitochondrial volume percentage per muscle, (**K**) intermyofibrillar mitochondrial volume percentage per muscle and (**L**) subsarcolemmal mitochondrial volume percentage per muscle; significance set at *p*-value of * *p* ≤ 0.050. Differences in EM parameters were assessed using unpaired two-sample trimmed mean test (20%) with 10,000-fold bootstrap.

Mean area (Figure 5A,B) as well as total intermyofibrillar (Figure 5C) were not different between both groups, while the total subsarcolemmal area was higher in PCS (Figure 5D). Groups neither differed in volume fraction nor the lengths of sarcomere/I-/A-Band (Figure 5E–I), whereas the mitochondrial volume percentage per muscle was higher in the total (Figure 5J) and subsarcolemmal area (Figure 5L) of PCS in comparison to CFS.

One-way MANOVA showed a significant difference between patients with CFS and patients with PCS (X^2^ (19) = 46.823, *p* < 0.001; Wilk’s Λ = 0.04876). All statistical evaluations of the electron microscopy analyses are provided in the sections *Intercorrelation between EM and oxidative OXPHOS parameters* in Supplementary Table S1 and *Group differences* (*bootstrapped results*) in Supplementary Table S2.

Correlation analysis

After observing differences in complex I activity, the subsarcolemmal and intermyofibrillar cristae score as well the peak oxygen consumption, a percentage bend correlation was performed to determine possible associations between these differing mitochondrial and performance parameters in the entire CFS and PCS study groups. Except for a positive correlation between the cristae score of subsarcolemmal mitochondria and their respective subsarcolemmal form, no further associations were observed (Table 2).

## 3. Discussion

In this study, functional as well as structural changes could be observed in the mitochondrial population of ME/CFS and PCS patients compared to age-matched unfit but otherwise healthy controls. Complex I activity was decreased in PCS and ME/CFS patients and more severely affected patients expressing lower activities of complex I and partly complex II (although not statistically significant) and more structural alterations in mitochondria.

While a functional impairment could be observed in PCS due to lower complex I activity, ME/CFS exhibited morphological changes in cristae and size, which might be a sign of detrimental effects by prolonged inactivity or immobility. Although the combined assessment of mitochondrial respiratory function and morphology does not allow for conclusions about the origin of respective mitochondrial dysfunction, the analyzed data may hint at a direct viral-induced chronic impact on mitochondrial function in PCS, separated from the detrimental morphological effects of ME/CFS, especially in the subsarcolemmal area.

Interestingly, more CFS patients than PCS (twelve vs. eight, respectively) could be exposed to the exercise test to determine the current performance level. This underlines the even more apparent fatigue symptoms in the PCS that prevent the respective participants from exerting even lighter physical loads. The underlying cause can only be speculated on, but less experience with fatigue (shorter time since disease onset; 35 vs. 20 months) or the observed mitochondrial function could be possible reasons. As there was no difference in muscle mass per kg between both patient groups, but a higher proportion of mitochondria in the skeletal muscle of PCS patients (especially in the subsarcolemmal area), the mitochondrial number of PCS per se seems unable to compensate for diminished aerobic exercise capacity determined by a cardiopulmonary exercise test.

Complex I activity was decreased in PCS due to still-unknown reasons. The most likely may be inflammation-induced dysfunction of transporters and functional mitochondrial complexes [25,26], but there is also an indication that mitochondrial genes are down-regulated during SARS-CoV-2 infection [27]. Further study results contributed additional data to this hypothesis: in PCS patients, histological differences with fewer capillaries, thicker capillary basement membranes and increased numbers of CD169^+^ macrophages as well as more complement system-related proteins in the serum were found [28]. Subsequently, these alterations potentially lead to immune-mediated structural changes in the microvasculature, potentially explaining activity-dependent fatigue and muscle pain [28]. ME/CFS patients also exhibited increased complement activation (especially C1q, C3, and C4 levels), which may even be used for clustering disease severity in this patient population itself [29], highlighting, once more, the similarities between PCS and ME/CFS conditions.

We did not separate subsarcolemmal and intermyofibrillar mitochondria before OXPHOS measurements, so the observed data reflect the OXPHOS capacity of the whole mitochondrial muscle population. Otherwise, there might be a difference only in one population of PCS (namely intermyofibrillar), as especially the subsarcolemmal mitochondria of CFS exhibited impaired cristae content, form, and size.

ME/CFS patients seem to exhibit a decreased mitochondrial function that positively correlates with disease severity. Booth et al. proposed a lack of essential substrates and partial blocking of the translocator protein sites in the mitochondria of ME/CFS patients [17], possibly contributing to less energy supply in these patients. If complex I, which is the entry point of NADH-reducing equivalents in the mitochondrial respiratory chain catalyzing the electron transport from NADH to ubiquinone, is only “insufficiently pumping” by impaired protein assembly, a logical consequence could be the observed diminished exercise capacity in PCS: the transmembrane potential, derived by complex I through coupling to the proton pump from the matrix to the intermembrane space, drives ATP formation by complex V [30], and the impaired ATP supply may result in the observed decreased performance.

In addition, a deficiency in complex I (at the bound flavine on the matrix side) and complex III (at the ubiquinol oxidation side) protein structure may contribute to increased reactive oxygen species levels (ROS), which might result in functional impairment of the whole electron-transporting machinery [31]. Unfortunately, we did not measure ROS concentrations in the skeletal muscle samples, which could have given further insight into the impact of a probable functional deficiency and mechanistic reasons.

The values obtained by electron microscopy reflect the current scientific evidence regarding the different morphology and function of the two populations. The subsarcolemmal mitochondria are usually located just below the sarcolemma and tend to be slightly larger, as observed in our study population. In contrast, while intermyofibrillar mitochondria are hardly affected by increased physical activity or inactivity, subsarcolemmal mitochondria seem to adapt strongly to external changes, both in morphology and in their respiratory capacity [32,33]. Subsarcolemmal mitochondria are also more sensitive than intermyofibrillar mitochondria to cell-damaging metabolites and external apoptosis signals [34,35].

In comparison, intermyofibrillar mitochondria are mostly embedded at the level of the Z-line in the contractile elements of skeletal muscle and are slightly smaller than subsarcolemmal mitochondria [32], as was also evident in our study population. However, they express significantly more oxidative phosphorylation-associated enzymes and have approximately 1.5-fold higher ATP synthesis capacity than subsarcolemmal mitochondria [36]. This suggests that intermyofibrillar mitochondria are subspecialized to meet the high energy demands of the contractile elements of muscle. The muscles of CFS patients exhibited smaller and more deformed subsarcolemmal mitochondria compared to PCS, assuming a prolonged detraining effect rather than a post-acute viral effect. Interestingly, PCS (although assessed only in a limited number of participants) exhibited lower muscle mass per kg body weight compared to CFS, confirming the assumption that a rather high detraining effect and prolonged inactivity were present. This could additionally contribute to the lower exercise capacity observed. Furthermore, there was no association determined by correlation analysis that showed any interdependence between mitochondrial function and aerobic capacity. This might propose another underlying cause of diminished performance in PCS in addition to mitochondrial dysfunctions, especially as there was no detrimental effects in the morphological area observed. A further approach might be to look at changes in mitochondrial volume density, as Hoppeler et al. showed in 1985 that the maximal oxygen consumption of an unit volume of mitochondria in muscle is close to 5 mL O_2_ min^1^ cm^−3^ in mammals, which implies that there is a constant volume of oxygen metabolized per unit volume of mitochondria and unit time that could give an estimation of the performance rate in working skeletal muscle tissue [37].

Colosio et al. examined post-COVID patients’ respiratory fitness and included muscle oxidative capacity measurements by near-infrared spectroscopy, histochemical analysis, O_2_ flux by high-resolution respirometry, and mitochondrial complex activities in skeletal muscle [38]. They found that post-COVID syndrome induced detrimental reductions in biomarkers for mitochondrial function (complex II mass specific oxygen flux), content, and biogenesis, as well as lower performance capacity determined by VO_2peak_ compared to recovered COVID controls. Although histochemical staining techniques were used instead of electron microscopy, these results confirm our observations of changes in muscular mitochondria regarding mitochondrial function and the performance of long-term-affected COVID-19 patients.

A general consideration that has to be taken into account is that only the oxygen consumption of mitochondria has been measured and, therefore, this cannot provide any information about the efficiency of the respiratory chain. In order to make statements about this hypothesis, data on the ATP/O ratio, H^+^/O ratio, or at least on the membrane potential would be necessary. However, determining these data is only possible with considerable effort, if at all. It is, therefore, quite conceivable that reduced mitochondrial respiration is compensated for by a higher efficiency and, therefore, has little functional effect.

### Strengths and Limitations

Due to the lack of an EM control group, the mean size and shape of CFS and PCS can only be compared between the respective syndromes. Nevertheless, the combined approach of both functional and morphological differences is rarely used in mitochondrial research and is a promising tool to support causal mechanistic theory building observed with both techniques. As there is no consensus to diagnose CFS accurately and separate it from fatigue caused by SARS-CoV-2 at the moment, the grouping of CFS and PCS was based on patient history and respective known viral infection. Although all CFS patients declared that symptoms were apparent before any SARS-CoV-2 infection (and PCS vice versa), it cannot be ruled out that an unknown infection might, nevertheless, have an impact on mitochondrial properties. Thus, the OXPHOS samples of the control group that were measured before the pandemic onset give valuable insights of the effects of viral infections on mitochondria.

## 4. Materials and Methods

### 4.1. Study Population

The Canadian Consensus Criteria (CCC according to Carruthers et al. [39]) for determining CFS were used to separate between patients suffering from CFS (10f/5m; age 36.1 ± 11.3 years; BMI 25.0 ± 3.4 kg/m^2^) and PCS (13f/2m; age 40.5 ± 12.8 years; BMI 21.9 ± 3.7 kg/m^2^). Healthy age-matched unfit individuals (N = 13, 7f/6m; age 36.2 ± 13.2 years; BMI 25.1 ± 4.5 kg/m^2^) were used as control group based on data from Buck et al. [40], where the following study protocol and measurements (muscle biopsy and OXPHOS) were carried out analogously as a previous study of our working group. Medical assessment as well as group classification of CFS and PCS were performed by two clinicians of the Division of Sports Medicine Ulm.

### 4.2. Study Design

**Figure 6:**
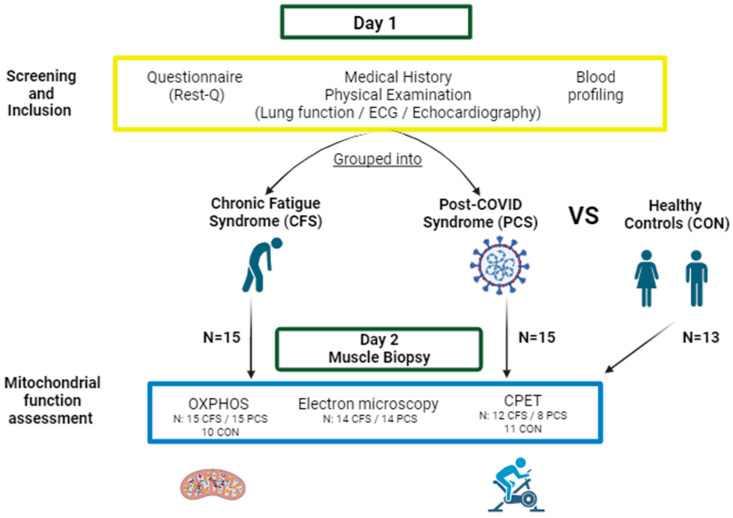
Schematic depiction of the study design. After presenting for a routine examination (Day 1), all participants were screened regarding their current disease status by fatigue-specific questionnaires, physical examinations, blood profiling and disease history. If participants met inclusion criteria, they were either grouped into CFS (chronic fatigue syndrome; 10f/5m) or PCS (post-COVID syndrome; 13f/2m). At a second examination (Day 2), oxidative phosphorylation capacity (OXPHOS), mitochondrial shape by electron microscopy, and a cardiopulmonary/spiroergometric exercise test (CPET) was conducted. OXPHOS, anthropometry, and CPX were compared to measurements of healthy control samples (7f/6m) (Figure designed with BioRender, https://www.biorender.com/, accessed on 24 January 2024).

The participants presented themselves in the out-patient clinic of the Sports Medicine facility of Ulm University Hospital with fatigue symptoms. All patients with presumed eligibility criteria were screened for either chronic fatigue or post-COVID-19 pathology. After giving informed consent, the participants’ patient history, height, as well as body composition by body impedance analysis (InBody 770, InBody Europe B.V., Eschborn, Germany) were determined. Muscle mass data for healthy controls were not available, as the measurement technique in the previous study was different. In addition, patients that were able to perform moderate to strenuous activities without post-exertional malaise performed a cardiopulmonary exercise test. An overview of the study procedure is presented in Figure 6.

### 4.3. Cardiopulmonary Exercise Test (CPET)

The CPET consisted of a standardized ramp exercise protocol until maximal exhaustion. After a warm-up of two minutes of unloaded pedaling, the power output was increased by 15 W/min (fatigue patient protocol depending on fitness and biological sex). Based on current symptoms, previous testing, or training status, the respective protocol was chosen to achieve total exhaustion within eight to twelve minutes. CPET was used to determine the relative maximal aerobic power (P_max_), displayed as W/kg body mass (BM), which was measured on a bicycle ergometer (Lode Corival and Lode Excalibur, Lode B.V., Groningen, The Netherlands), and the peak oxygen consumption VO_2peak_, displayed as mL/min/kg BM.

After the CPET, participants were released after further routine examinations. A skeletal muscle biopsy as well as subsequent OXPHOS and EM measurements were conducted on a separate day at a second examination.

### 4.4. Skeletal Muscle Biopsy Sampling

Sampling of skeletal muscle was performed using the Bergström biopsy technique according to published protocols [41]. Participants’ muscle samples were obtained under local anaesthesia from the *M. quadriceps femoris* of the dominant side of the participants, approximately 20 cm above the knee.

For RNA isolation, muscle tissue was incubated for 24 h with RNAlater (QIAGEN GmbH, Hilden, Germany) at 4 °C and then stored in cryotubes at −80 °C until further analysis. Muscle tissue for protein examination was immediately cryopreserved with liquid nitrogen and stored at −80 °C until further analysis.

For electron microscopy, around 50 mg of muscle tissue was stabilized and fixed in a solution of 2.5% glutaraldehyde, 2% paraformaldehyde, 1% saccharose, and 0.1 M phosphate buffer and stored at 4 °C until further processing.

For respirometry with the Oxymeter Oroboros^®^ Oxygraph-2K (Oroboros Instruments, Innsbruck, Austria), 50 mg of muscle sample was stabilized in CUSTODIOL solution (Dr. Franz Köhler Chemie GmbH, Bensheim, Germany) and stored at 4 °C until analysis.

### 4.5. Measurement of Mitochondrial Respiration

The detailed experimental procedures can be found in the manuscript of Buck et al. [40]. In short, measurements of skeletal muscle biopsy samples were carried out simultaneously in both chambers of the Oxymeter Oroboros^®^ Oxygraph-2K. Oxygen consumption coupled to ATP synthase was determined in one chamber, and oxygen consumption under uncoupled conditions was determined in the other chamber. The MiRO5 cell medium (Oroboros Instruments, Innsbruck, Austria) was used as a mitochondrial respiration medium according to the manufacturer’s instructions. After prior rinsing with 70% ethanol and distilled water, the two chambers were each filled with approximately 4–5 mg of the muscle tissue and 2 mL of the cooled MiRO5 cell medium. The volume of the chambers was adjusted accordingly to 2 mL and a temperature of 37 °C was set on the Peltier display. Calibration of the device was completed when the oxygen flow was 0 pmol/(s × mg).

Every sample was measured in duplicates and normalized to the amount of tissue per chamber. The titration sequence used for the human muscle samples was as follows: 10 mM pyruvate, 5mM malate, 10 mM glutamate, 5 mM ADP, 10 μM cytochrome c, 1 mM octanoyl carnitine, 10 mM succinate 5 μM oligomycin, 0.5 μM carbonyl cyanide p-(trifluoromethoxy)-phenylhydrazone (FCCP), 0.5 μM rotenone, and 5 μM antimycin A. The respective oxygen flux was analyzed by the Oroboros^®^ system.

### 4.6. Electron Micrography of Mitochondrial Form and Cristae

Microscopy of the tissue samples took place in the Department of Electron Microscopy at the University of Ulm. The acquisition of the electron microscopic images and their further analysis were blinded at all time points. All images were acquired with a transmission electron microscope (TEM) (JEM-1400, Jeol, Tokyo, Japan) equipped with a CCD camera.

To ensure the most standardized and randomized selection of image sections, five image sections were selected within a preparation using the grid pattern of the nickel mesh grid on which the tissue sections were placed. If the muscle sections showed an irregular shape, the next possible grid was used. Within these image sections, 20 images were taken of each of the preparations at 40,000× magnification. Within each of the preparations, ten subsarcolemmal and ten intermyofibrillar regions were selected to represent as balanced a distribution of the different mitochondrial types as possible. Thus, in each of the selected grid sections, four image acquisitions of skeletal muscle were made, two each of subsarcolemmal and two of intermyofibrillar skeletal muscle sections. Image sections in which the structure of the skeletal muscle cells could not be clearly identified due to artefacts were not used.

### 4.7. Evaluation of the Image Recordings with ImageJ

The image processing program Image J version 1.53t was used for quantitative evaluation of the electron microscopic images. The evaluation program was used to examine the following parameters:

(a) Average length, width and circumference of mitochondria;
(b) Shape of mitochondria (0 = inconspicuous, 1 = slightly changed shape, 2 = strongly changed shape, 3 = damage of mitochondrial membrane);
(c) Average volume of mitochondria;
(d) Volume fraction of mitochondria in skeletal muscle;
(e) Surface area fraction of cristae;
(f) Volume density of cristae (= cristae surface area/area of mitochondria);
(g) Cristae score (0 = no sharply defined cristae, 1 = more than 50% the mitochondrial volume without cristae, 2 = more than 25% the mitochondrial volume without cristae, 3 = less than 25% the mitochondrial volume without cristae but cristae irregular, 4 = less than 25% the mitochondrial volume without cristae and regular cristae).

### 4.8. Statistical Analysis

Data analyses were performed using R version 4.2.2 [42]. Robust one-way ANOVAs were conducted to assess the differences in the mitochondrial activity between healthy controls, patients with CFS, and patients with PCS. Post hoc tests were applied based on robust linear regression models (R-package: MASS, [43]) with *p*-value-adjustment according to Holm. Differences in EM parameters were assessed using unpaired two-sample trimmed mean test (20%) with 10,000-fold bootstrap [42]. The associations between the assessed mitochondrial parameters as well as between CPET and mitochondrial parameters were analyzed using percentage bend correlation with fdr correction (false-discovery rate according to [44]). Due to high correlations between the mitochondrial parameters, a multivariate analysis via a robust MANOVA (R-package: MASS) was performed.

## 5. Conclusions

Although CFS and PCS exhibit increased fatigue and mitochondrial capacity is impaired in both populations, the progressed pathological morphological alterations in CFS suggest structural changes due to prolonged inactivity or unknown molecular causes like increased ROS production compared to the probably direct virus-induced changes in PCS. Although the decreased OXPHOS capacity in PCS per se may be a result of disease-caused physical inactivity, the significantly lower complex I activity suggests a treatment target in this population due to its first role in the electron gradient machinery and importance in energy production. The increased morphological changes in CFS in the subsarcolemmal area suggest long-term adaptation processes, whereas changes in PCS mitochondrial function seem to be related to a direct impact of the virus.

## Figures and Tables

**Table 1:** Overview of clinically collected data of the study participants. Significance set at * *p* < 0.050. Results based on robust one-way ANOVAs comparison between healthy controls, CFS and PCS. Post hoc tests were applied based on robust linear regression models with *p*-value adjustment according to Holm.

	HC (7f/6m)			CFS (10f/5m)			PCS (13f/2m)				
	M	SD	Median	M	SD	Median	M	SD	Median	t	*p*
Age	36.23	13.17	32.00	36.13	11.65	33.50	40.47	13.21	38.00	0.56	0.5762
Body mass (kg)	76.87	17.68	78.15	76.43	12.72	73.10	62.58	15.85	59.80	3.85	0.0302 *
Body height (cm)	173.92	12.06	175.00	174.07	10.37	172.90	169.07	12.06	175.00	1.20	0.3129
Body mass index (kg/m^2^)	25.10	4.51	24.45	25.04	3.57	24.30	21.85	3.85	21.30	3.07	0.0586
Body fat (%)	21.03	4.44	21.55	32.92	8.33	32.95	22.87	7.02	21.55	5.57	0.0114 *
Absolute VO_2peak_ (L/min)	2.35	0.55	2.36	1.94	0.94	1.75	1.36	0.71	1.25	4.13	0.0265 *
Relative VO_2peak_ (mL/min/kg)	30.94	4.87	33.00	24.97	9.93	22.46	21.65	6.93	22.36	3.90	0.0317 *
CCC-Score				19.00	5.91	17.50	18.54	7.11	18.00	0.03	0.8621
Time since disease diagnosis (months)				35.20	44.06	23.00	20.47	15.56	15.00	1.49	0.2322
Muscle mass (%)				29.28	13.41	32.72	39.80	8.27	40.88	4.29	0.0550

Abbreviations: VO_2peak_—maximum oxygen uptake; CCC score—value obtained in the Canadian Consensus Criteria (CCC) questionnaire; M—mean value; SD—standard deviation; HC—healthy control group; CFS—chronic fatigue syndrome patients; PCS—post-COVID syndrome patients.

**Table 2:** Correlation analysis results for function-related mitochondrial parameters and patients’ performance status. There was only a correlation between the subsarcolemmal cristae score and form. The association between the assessed mitochondrial parameters as well as between CPET and mitochondrial parameters were analyzed using percentage bend correlation with fdr correction. N: All available data of participants of CFS and PCS that were integrated into the statistical analysis.

Parameter 1	Parameter 2	r	CI	CI_Low	CI_High	t	*p*	N
VO_2__peak	CriSco_I_m	−0.27	0.95	−0.65	0.23	−1.11	0.42	18
VO_2__peak	CriSco_S_m	0.08	0.95	−0.40	0.53	0.31	0.82	18
VO_2__peak	Form_S_m	0.41	0.95	−0.07	0.73	1.78	0.28	18
VO_2__peak	Form_I_m	0.04	0.95	−0.44	0.50	0.16	0.88	18
VO_2__peak	CA_I.x	0.23	0.95	−0.16	0.56	1.20	0.40	27
CriSco_I_m	CriSco_S_m	0.70	0.95	0.43	0.85	4.86	0.00	27
CriSco_I_m	Form_S_m	0.35	0.95	−0.04	0.64	1.85	0.28	27
CriSco_I_m	Form_I_m	0.26	0.95	−0.13	0.58	1.35	0.40	27
CriSco_I_m	CA_I.x	−0.11	0.95	−0.47	0.28	−0.54	0.68	27
CriSco_S_m	Form_S_m	0.51	0.95	0.16	0.74	2.94	**0.05**	27
CriSco_S_m	Form_I_m	0.24	0.95	−0.15	0.57	1.24	0.40	27
CriSco_S_m	CA_I.x	−0.27	0.95	−0.59	0.12	−1.41	0.40	27
Form_S_m	Form_I_m	0.38	0.95	0.00	0.67	2.08	0.24	27
Form_S_m	CA_I.x	−0.16	0.95	−0.51	0.24	−0.80	0.54	27
Form_I_m	CA_I.x	0.20	0.95	−0.19	0.54	1.05	0.42	27

Abbreviations: VO_2peak_: Peak oxygen consumption; CriSco_I_m: Cristae score of intermyofibrillar mitochondria; CriSco_S_m: Cristae score of subsarcolemmal mitochondria; Form_I_m: Form of intermyofibrillar mitochondria; Form_S_m: Form of subsarcolemmal mitochondria; CA_I.x: Complex I activity.

## Data Availability

All data which are not provided in the main manuscript or Supplementary Files can be provided by the corresponding author on reasonable request. Not all data are publicly available due to privacy restrictions.

## Supplementary Materials

The supporting information can be downloaded at: https://www.mdpi.com/article/10.3390/ijms25031675/s1.
