# Supplementary material for: Functional and Morphological Differences of Muscle Mitochondria in Chronic Fatigue Syndrome and Post-COVID Syndrome
Source: Int J Mol Sci. 2024 Jan 30;25(3):1675. doi: 10.3390/ijms25031675 (PMC10855807; doi:10.3390/ijms25031675)
Supplement: Supplementary file 1 [file ijms-25-01675-s001.zip › Supplementary Material_Table S1 + S2.pdf]

Supplementary Table S1

Intercorrelation between electron microscopy (EM) and oxidative phosphorylation (OXPHOS) parameters. All statistical parameters are included.

MA_Coupled: Maximal Activity coupled; MA_Uncoupled: Maximal Activity uncoupled; CA_I Complex I Activity; CA_II Complex II Activity; CA_IV Complex IV Activity; Form_I_m: Form of intermyofibrillar mitochondria; Form_S_m Form of subsarcolemmal mitochondria: CriSco_I_m: Cristae score intermyofibrillar mitochondria; CriSco_S_m: Cristae score of subsarcolemmal mitochondria; smD_I_m: smallest diameter intermyofibrillar mitochondria; smD_S_m: smallest diameter subsarcolemmal mitochondria; meD_I_m: mean diameter intermyofibrillar mitochondria; meD_S_m: mean diameter subsarcolemmal mitochondria; Dia_I_m: perimeter intermyofibrillar mitochondria; Dia_S_m: perimeter subsarcolemmal mitochondria; AreaMito_I_m: area intermyofibrillar mitochondria; AreaMito_S_m: area subsarcolemmal mitochondria; AreaMitoTotal_I_m: area of all intermyofibrillar mitochondria; AreaMitoTotal_S_m: area of all subsarcolemmal mitochondria; Vol_I_m: volume intermyofibrillar mitochondria; Vol_S_m: volume subsarcolemmal mitochondria; Sar_I_m: sarcomere length muscle; IB_I_m: I-band length muscle; AB_I_m: A-band length muscle; Mito_Vol%_Mus_Total: volume percent of mitochondria in all muscle; Mito_Vol%_Mus_I: volume percent of mitochondria in all intermyofibrillar muscle; Mito_Vol%_Mus_S: volume percent of mitochondria in subsarcolemmal muscle

Parameter 1	Parameter 2	r	CI_low	CI_high	t	df	N	p	*p* fdr
MA_Coupled	MA_Uncoupled	0.975	0.952	0.987	26.622	37	39	0.000	0.000
MA_Coupled	CA_I	0.865	0.755	0.927	10.477	37	39	0.000	0.000
MA_Coupled	CA_II	0.834	0.706	0.909	9.319	38	40	0.000	0.000
MA_Coupled	CA_IV	0.839	0.714	0.912	9.503	38	40	0.000	0.000
MA_Coupled	Form_I_m	0.198	-0.197	0.537	1.007	25	27	0.323	0.535
MA_Coupled	Form_S_m	0.010	-0.371	0.389	0.050	25	27	0.960	0.977
MA_Coupled	CriSco_I_m	-0.091	-0.456	0.299	-0.459	25	27	0.650	0.806
MA_Coupled	CriSco_S_m	-0.287	-0.602	0.104	-1.500	25	27	0.146	0.367
MA_Coupled	smD_I_m	0.022	-0.361	0.399	0.110	25	27	0.913	0.948
MA_Coupled	smD_S_m	-0.053	-0.425	0.334	-0.266	25	27	0.792	0.895
MA_Coupled	meD_I_m	-0.026	-0.402	0.357	-0.130	25	27	0.897	0.943
MA_Coupled	meD_S_m	-0.134	-0.489	0.259	-0.675	25	27	0.506	0.690
MA_Coupled	Dia_I_m	0.084	-0.306	0.450	0.422	25	27	0.676	0.824
MA_Coupled	Dia_S_m	0.000	-0.380	0.380	0.000	25	27	1.000	1.000
MA_Coupled	AreaMito_I_m	-0.043	-0.416	0.342	-0.217	25	27	0.830	0.905
MA_Coupled	AreaMito_S_m	-0.177	-0.522	0.218	-0.897	25	27	0.378	0.567
MA_Coupled	AreaMitoTotal_I_m	0.049	-0.337	0.421	0.246	25	27	0.807	0.895
MA_Coupled	AreaMitoTotal_S_m	-0.342	-0.639	0.044	-1.820	25	27	0.081	0.248
MA_Coupled	Vol_I_m	-0.185	-0.528	0.210	-0.941	25	27	0.356	0.554
MA_Coupled	Vol_S_m	-0.344	-0.640	0.042	-1.830	25	27	0.079	0.246
MA_Coupled	Sar_I_m	0.029	-0.355	0.404	0.144	25	27	0.887	0.940
MA_Coupled	IB_I_m	0.230	-0.164	0.561	1.182	25	27	0.248	0.486
MA_Coupled	AB_I_m	-0.023	-0.399	0.361	-0.113	25	27	0.911	0.948
MA_Coupled	Mito_Vol%_Mus_Total	-0.333	-0.633	0.054	-1.767	25	27	0.089	0.271
MA_Coupled	Mito_Vol%_Mus_I	-0.185	-0.528	0.210	-0.941	25	27	0.356	0.554
MA_Coupled	Mito_Vol%_Mus_S	-0.344	-0.640	0.042	-1.830	25	27	0.079	0.246
MA_Uncoupled	CA_I	0.893	0.802	0.943	11.894	36	38	0.000	0.000
MA_Uncoupled	CA_II	0.807	0.660	0.895	8.327	37	39	0.000	0.000
MA_Uncoupled	CA_IV	0.814	0.671	0.899	8.520	37	39	0.000	0.000

Parameter 1	Parameter 2	r	CI_low	CI_high	t	df	N	p	p_{fdr}
MA_Uncoupled	Form_I_m	0.122	-0.278	0.486	0.602	24	26	0.553	0.717
MA_Uncoupled	Form_S_m	-0.120	-0.485	0.280	-0.594	24	26	0.558	0.717
MA_Uncoupled	CriSco_I_m	-0.117	-0.482	0.283	-0.576	24	26	0.570	0.730
MA_Uncoupled	CriSco_S_m	-0.317	-0.628	0.080	-1.639	24	26	0.114	0.321
MA_Uncoupled	smD_I_m	-0.053	-0.432	0.341	-0.262	24	26	0.796	0.895
MA_Uncoupled	smD_S_m	-0.095	-0.465	0.304	-0.467	24	26	0.645	0.802
MA_Uncoupled	meD_I_m	-0.123	-0.487	0.278	-0.607	24	26	0.550	0.717
MA_Uncoupled	meD_S_m	-0.189	-0.537	0.214	-0.945	24	26	0.354	0.554
MA_Uncoupled	Dia_I_m	0.019	-0.371	0.403	0.091	24	26	0.928	0.961
MA_Uncoupled	Dia_S_m	-0.030	-0.412	0.362	-0.146	24	26	0.885	0.940
MA_Uncoupled	AreaMito_I_m	-0.151	-0.509	0.251	-0.748	24	26	0.462	0.656
MA_Uncoupled	AreaMito_S_m	-0.242	-0.575	0.161	-1.220	24	26	0.234	0.472
MA_Uncoupled	AreaMitoTotal_I_m	0.013	-0.376	0.398	0.064	24	26	0.949	0.971
MA_Uncoupled	AreaMitoTotal_S_m	-0.351	-0.650	0.042	-1.839	24	26	0.078	0.246
MA_Uncoupled	Vol_I_m	-0.249	-0.580	0.154	-1.257	24	26	0.221	0.461
MA_Uncoupled	Vol_S_m	-0.354	-0.652	0.039	-1.852	24	26	0.076	0.246
MA_Uncoupled	Sar_I_m	-0.068	-0.444	0.328	-0.334	24	26	0.741	0.858
MA_Uncoupled	IB_I_m	0.232	-0.170	0.569	1.170	24	26	0.253	0.486
MA_Uncoupled	AB_I_m	-0.143	-0.503	0.258	-0.709	24	26	0.485	0.681
MA_Uncoupled	Mito_Vol%_Mus_Total	-0.360	-0.656	0.032	-1.892	24	26	0.071	0.236
MA_Uncoupled	Mito_Vol%_Mus_I	-0.247	-0.579	0.156	-1.247	24	26	0.224	0.463
MA_Uncoupled	Mito_Vol%_Mus_S	-0.354	-0.652	0.039	-1.852	24	26	0.076	0.246
CA_I	CA_II	0.682	0.467	0.821	5.671	37	39	0.000	0.000
CA_I	CA_IV	0.655	0.428	0.804	5.271	37	39	0.000	0.000
CA_I	Form_I_m	0.205	-0.190	0.543	1.047	25	27	0.305	0.518
CA_I	Form_S_m	-0.157	-0.507	0.237	-0.796	25	27	0.434	0.624
CA_I	CriSco_I_m	-0.108	-0.468	0.284	-0.541	25	27	0.594	0.755
CA_I	CriSco_S_m	-0.271	-0.590	0.122	-1.406	25	27	0.172	0.403
CA_I	smD_I_m	0.058	-0.329	0.428	0.289	25	27	0.775	0.886
CA_I	smD_S_m	0.024	-0.359	0.400	0.121	25	27	0.905	0.948
CA_I	meD_I_m	0.078	-0.311	0.445	0.390	25	27	0.700	0.833
CA_I	meD_S_m	-0.104	-0.466	0.287	-0.524	25	27	0.605	0.761
CA_I	Dia_I_m	0.187	-0.208	0.529	0.951	25	27	0.351	0.554
CA_I	Dia_S_m	0.046	-0.340	0.418	0.228	25	27	0.821	0.904
CA_I	AreaMito_I_m	0.003	-0.377	0.383	0.016	25	27	0.987	0.999
CA_I	AreaMito_S_m	-0.080	-0.447	0.309	-0.403	25	27	0.690	0.830
CA_I	AreaMitoTotal_I_m	0.097	-0.294	0.460	0.487	25	27	0.631	0.788
CA_I	AreaMitoTotal_S_m	-0.242	-0.569	0.152	-1.246	25	27	0.224	0.463
CA_I	Vol_I_m	-0.125	-0.482	0.267	-0.632	25	27	0.533	0.706
CA_I	Vol_S_m	-0.252	-0.577	0.142	-1.301	25	27	0.205	0.442

Parameter 1	Parameter 2	r	CI_low	CI_high	t	df	N	p	*p* fdr
CA_I	Sar_I_m	0.180	-0.214	0.524	0.917	25	27	0.368	0.560
CA_I	IB_I_m	0.385	0.006	0.668	2.088	25	27	0.047	0.172
CA_I	AB_I_m	0.162	-0.233	0.510	0.819	25	27	0.420	0.612
CA_I	Mito_Vol%_Mus_Total	-0.229	-0.561	0.165	-1.178	25	27	0.250	0.486
CA_I	Mito_Vol%_Mus_I	-0.128	-0.485	0.265	-0.646	25	27	0.524	0.702
CA_I	Mito_Vol%_Mus_S	-0.252	-0.577	0.142	-1.301	25	27	0.205	0.442
CA_II	CA_IV	0.700	0.497	0.830	6.043	38	40	0.000	0.000
CA_II	Form_I_m	0.293	-0.098	0.606	1.534	25	27	0.138	0.363
CA_II	Form_S_m	0.131	-0.262	0.487	0.660	25	27	0.516	0.693
CA_II	CriSco_I_m	0.001	-0.379	0.381	0.004	25	27	0.997	1.000
CA_II	CriSco_S_m	-0.188	-0.530	0.206	-0.959	25	27	0.347	0.554
CA_II	smD_I_m	0.049	-0.337	0.421	0.245	25	27	0.808	0.895
CA_II	smD_S_m	-0.012	-0.390	0.370	-0.058	25	27	0.954	0.974
CA_II	meD_I_m	0.067	-0.321	0.436	0.337	25	27	0.739	0.858
CA_II	meD_S_m	-0.028	-0.404	0.356	-0.141	25	27	0.889	0.940
CA_II	Dia_I_m	0.111	-0.280	0.472	0.561	25	27	0.580	0.740
CA_II	Dia_S_m	0.030	-0.354	0.405	0.148	25	27	0.883	0.940
CA_II	AreaMito_I_m	-0.002	-0.382	0.378	-0.010	25	27	0.992	1.000
CA_II	AreaMito_S_m	-0.106	-0.467	0.286	-0.531	25	27	0.600	0.760
CA_II	AreaMitoTotal_I_m	0.068	-0.320	0.437	0.340	25	27	0.736	0.858
CA_II	AreaMitoTotal_S_m	-0.219	-0.553	0.176	-1.121	25	27	0.273	0.494
CA_II	Vol_I_m	-0.180	-0.524	0.215	-0.915	25	27	0.369	0.560
CA_II	Vol_S_m	-0.220	-0.553	0.175	-1.126	25	27	0.271	0.494
CA_II	Sar_I_m	0.083	-0.307	0.449	0.416	25	27	0.681	0.825
CA_II	IB_I_m	0.044	-0.342	0.417	0.221	25	27	0.827	0.904
CA_II	AB_I_m	0.087	-0.303	0.452	0.436	25	27	0.667	0.815
CA_II	Mito_Vol%_Mus_Total	-0.228	-0.559	0.167	-1.170	25	27	0.253	0.486
CA_II	Mito_Vol%_Mus_I	-0.179	-0.523	0.216	-0.910	25	27	0.371	0.560
CA_II	Mito_Vol%_Mus_S	-0.220	-0.553	0.175	-1.126	25	27	0.271	0.494
CA_IV	Form_I_m	0.163	-0.232	0.511	0.825	25	27	0.417	0.612
CA_IV	Form_S_m	-0.016	-0.394	0.366	-0.081	25	27	0.936	0.963
CA_IV	CriSco_I_m	-0.131	-0.487	0.262	-0.662	25	27	0.514	0.693
CA_IV	CriSco_S_m	-0.269	-0.589	0.123	-1.398	25	27	0.174	0.405
CA_IV	smD_I_m	0.016	-0.367	0.393	0.078	25	27	0.939	0.963
CA_IV	smD_S_m	-0.053	-0.425	0.333	-0.267	25	27	0.792	0.895
CA_IV	meD_I_m	-0.072	-0.440	0.316	-0.363	25	27	0.720	0.848
CA_IV	meD_S_m	-0.083	-0.449	0.307	-0.415	25	27	0.682	0.825
CA_IV	Dia_I_m	0.008	-0.373	0.387	0.042	25	27	0.967	0.980
CA_IV	Dia_S_m	0.028	-0.356	0.404	0.141	25	27	0.889	0.940
CA_IV	AreaMito_I_m	-0.052	-0.424	0.335	-0.261	25	27	0.796	0.895

Parameter 1	Parameter 2	r	CI_low	CI_high	t	df	N	p	p_{fdr}
CA_IV	AreaMito_S_m	-0.137	-0.491	0.257	-0.691	25	27	0.496	0.683
CA_IV	AreaMitoTotal_I_m	-0.124	-0.481	0.269	-0.625	25	27	0.538	0.707
CA_IV	AreaMitoTotal_S_m	-0.274	-0.593	0.118	-1.426	25	27	0.166	0.397
CA_IV	Vol_I_m	-0.189	-0.531	0.206	-0.962	25	27	0.345	0.554
CA_IV	Vol_S_m	-0.275	-0.593	0.117	-1.432	25	27	0.165	0.396
CA_IV	Sar_I_m	-0.001	-0.381	0.379	-0.006	25	27	0.995	1.000
CA_IV	IB_I_m	0.262	-0.131	0.584	1.359	25	27	0.186	0.420
CA_IV	AB_I_m	-0.105	-0.466	0.286	-0.528	25	27	0.602	0.760
CA_IV	Mito_Vol%_Mus_Total	-0.280	-0.596	0.112	-1.456	25	27	0.158	0.390
CA_IV	Mito_Vol%_Mus_I	-0.186	-0.529	0.209	-0.947	25	27	0.353	0.554
CA_IV	Mito_Vol%_Mus_S	-0.275	-0.593	0.117	-1.432	25	27	0.165	0.396
Form_I_m	Form_S_m	0.384	0.005	0.667	2.080	25	27	0.048	0.173
Form_I_m	CriSco_I_m	0.260	-0.133	0.582	1.346	25	27	0.190	0.420
Form_I_m	CriSco_S_m	0.241	-0.153	0.569	1.241	25	27	0.226	0.464
Form_I_m	smD_I_m	0.228	-0.166	0.560	1.172	25	27	0.252	0.486
Form_I_m	smD_S_m	0.152	-0.242	0.503	0.769	25	27	0.449	0.641
Form_I_m	meD_I_m	0.274	-0.119	0.592	1.423	25	27	0.167	0.397
Form_I_m	meD_S_m	0.240	-0.154	0.568	1.237	25	27	0.228	0.465
Form_I_m	Dia_I_m	0.236	-0.158	0.565	1.215	25	27	0.236	0.473
Form_I_m	Dia_S_m	0.218	-0.177	0.552	1.116	25	27	0.275	0.494
Form_I_m	AreaMito_I_m	0.169	-0.226	0.516	0.857	25	27	0.400	0.596
Form_I_m	AreaMito_S_m	0.209	-0.186	0.545	1.066	25	27	0.297	0.515
Form_I_m	AreaMitoTotal_I_m	0.221	-0.174	0.554	1.132	25	27	0.268	0.494
Form_I_m	AreaMitoTotal_S_m	-0.040	-0.414	0.345	-0.201	25	27	0.842	0.915
Form_I_m	Vol_I_m	0.490	0.135	0.734	2.812	25	27	0.009	0.041
Form_I_m	Vol_S_m	-0.049	-0.421	0.337	-0.247	25	27	0.807	0.895
Form_I_m	Sar_I_m	0.203	-0.192	0.541	1.037	25	27	0.310	0.518
Form_I_m	IB_I_m	-0.067	-0.436	0.321	-0.336	25	27	0.740	0.858
Form_I_m	AB_I_m	0.216	-0.178	0.551	1.107	25	27	0.279	0.494
Form_I_m	Mito_Vol%_Mus_Total	0.079	-0.311	0.445	0.395	25	27	0.696	0.832
Form_I_m	Mito_Vol%_Mus_I	0.492	0.138	0.735	2.825	25	27	0.009	0.040
Form_I_m	Mito_Vol%_Mus_S	-0.049	-0.421	0.337	-0.247	25	27	0.807	0.895
Form_S_m	CriSco_I_m	0.347	-0.038	0.643	1.852	25	27	0.076	0.246
Form_S_m	CriSco_S_m	0.507	0.158	0.744	2.945	25	27	0.007	0.032
Form_S_m	smD_I_m	-0.087	-0.452	0.303	-0.438	25	27	0.665	0.815
Form_S_m	smD_S_m	0.088	-0.302	0.453	0.442	25	27	0.662	0.815
Form_S_m	meD_I_m	-0.039	-0.413	0.346	-0.196	25	27	0.846	0.916
Form_S_m	meD_S_m	0.262	-0.131	0.584	1.360	25	27	0.186	0.420

Parameter 1	Parameter 2	r	CI_low	CI_high	t	df	N	p	*p* fdr
Form_S_m	Dia_I_m	-0.162	-0.510	0.232	-0.820	25	27	0.420	0.612
Form_S_m	Dia_S_m	0.133	-0.260	0.488	0.670	25	27	0.509	0.690
Form_S_m	AreaMito_I_m	-0.140	-0.493	0.254	-0.705	25	27	0.487	0.681
Form_S_m	AreaMito_S_m	0.203	-0.191	0.542	1.039	25	27	0.309	0.518
Form_S_m	AreaMitoTotal_I_m	0.316	-0.072	0.622	1.667	25	27	0.108	0.316
Form_S_m	AreaMitoTotal_S_m	0.218	-0.176	0.552	1.118	25	27	0.274	0.494
Form_S_m	Vol_I_m	0.121	-0.271	0.479	0.611	25	27	0.547	0.716
Form_S_m	Vol_S_m	0.216	-0.178	0.551	1.108	25	27	0.278	0.494
Form_S_m	Sar_I_m	0.185	-0.210	0.528	0.943	25	27	0.355	0.554
Form_S_m	IB_I_m	-0.023	-0.399	0.360	-0.115	25	27	0.910	0.948
Form_S_m	AB_I_m	0.247	-0.147	0.573	1.274	25	27	0.214	0.456
Form_S_m	Mito_Vol%_Mus_Total	0.133	-0.260	0.488	0.670	25	27	0.509	0.690
Form_S_m	Mito_Vol%_Mus_I	0.119	-0.274	0.477	0.598	25	27	0.555	0.717
Form_S_m	Mito_Vol%_Mus_S	0.216	-0.178	0.551	1.108	25	27	0.278	0.494
CriSco_I_m	CriSco_S_m	0.697	0.431	0.851	4.857	25	27	0.000	0.000
CriSco_I_m	smD_I_m	0.073	-0.316	0.441	0.367	25	27	0.717	0.847
CriSco_I_m	smD_S_m	0.164	-0.230	0.512	0.831	25	27	0.414	0.610
CriSco_I_m	meD_I_m	0.267	-0.126	0.587	1.384	25	27	0.178	0.412
CriSco_I_m	meD_S_m	0.277	-0.115	0.594	1.440	25	27	0.162	0.396
CriSco_I_m	Dia_I_m	0.159	-0.235	0.508	0.805	25	27	0.429	0.619
CriSco_I_m	Dia_S_m	0.161	-0.233	0.510	0.815	25	27	0.423	0.613
CriSco_I_m	AreaMito_I_m	0.189	-0.205	0.531	0.964	25	27	0.344	0.554
CriSco_I_m	AreaMito_S_m	0.244	-0.150	0.571	1.256	25	27	0.221	0.461
CriSco_I_m	AreaMitoTotal_I_m	0.222	-0.172	0.556	1.141	25	27	0.265	0.494
CriSco_I_m	AreaMitoTotal_S_m	0.231	-0.164	0.562	1.186	25	27	0.247	0.486
CriSco_I_m	Vol_I_m	0.348	-0.037	0.643	1.856	25	27	0.075	0.246
CriSco_I_m	Vol_S_m	0.224	-0.171	0.557	1.149	25	27	0.262	0.494
CriSco_I_m	Sar_I_m	0.203	-0.192	0.541	1.036	25	27	0.310	0.518
CriSco_I_m	IB_I_m	0.059	-0.329	0.429	0.295	25	27	0.771	0.884
CriSco_I_m	AB_I_m	0.261	-0.132	0.583	1.352	25	27	0.188	0.420
CriSco_I_m	Mito_Vol%_Mus_Total	0.228	-0.166	0.560	1.172	25	27	0.252	0.486
CriSco_I_m	Mito_Vol%_Mus_I	0.342	-0.044	0.639	1.817	25	27	0.081	0.248
CriSco_I_m	Mito_Vol%_Mus_S	0.224	-0.171	0.557	1.149	25	27	0.262	0.494
CriSco_S_m	smD_I_m	-0.137	-0.491	0.256	-0.691	25	27	0.496	0.683
CriSco_S_m	smD_S_m	0.168	-0.226	0.516	0.855	25	27	0.401	0.596
CriSco_S_m	meD_I_m	0.027	-0.356	0.403	0.137	25	27	0.892	0.941
CriSco_S_m	meD_S_m	0.320	-0.068	0.624	1.688	25	27	0.104	0.306
CriSco_S_m	Dia_I_m	-0.077	-0.444	0.313	-0.384	25	27	0.704	0.835

Parameter 1	Parameter 2	r	CI_low	CI_high	t	df	N	p	p_{fdr}
CriSco_S_m	Dia_S_m	0.238	-0.156	0.567	1.224	25	27	0.233	0.472
CriSco_S_m	AreaMito_I_m	-0.072	-0.440	0.317	-0.359	25	27	0.722	0.848
CriSco_S_m	AreaMito_S_m	0.282	-0.110	0.598	1.468	25	27	0.154	0.385
CriSco_S_m	AreaMitoTotal_I_m	0.206	-0.189	0.543	1.050	25	27	0.304	0.518
CriSco_S_m	AreaMitoTotal_S_m	0.473	0.113	0.723	2.681	25	27	0.013	0.055
CriSco_S_m	Vol_I_m	0.146	-0.248	0.498	0.738	25	27	0.468	0.662
CriSco_S_m	Vol_S_m	0.471	0.111	0.722	2.673	25	27	0.013	0.055
CriSco_S_m	Sar_I_m	0.295	-0.096	0.607	1.542	25	27	0.136	0.363
CriSco_S_m	IB_I_m	-0.031	-0.406	0.353	-0.154	25	27	0.879	0.940
CriSco_S_m	AB_I_m	0.408	0.034	0.682	2.237	25	27	0.034	0.130
CriSco_S_m	Mito_Vol%_Mus_Total	0.405	0.029	0.680	2.212	25	27	0.036	0.134
CriSco_S_m	Mito_Vol%_Mus_I	0.138	-0.255	0.492	0.698	25	27	0.492	0.682
CriSco_S_m	Mito_Vol%_Mus_S	0.471	0.111	0.722	2.673	25	27	0.013	0.055
smD_I_m	smD_S_m	0.804	0.610	0.907	6.760	25	27	0.000	0.000
smD_I_m	meD_I_m	0.906	0.803	0.957	10.730	25	27	0.000	0.000
smD_I_m	meD_S_m	0.655	0.365	0.829	4.329	25	27	0.000	0.001
smD_I_m	Dia_I_m	0.865	0.723	0.937	8.628	25	27	0.000	0.000
smD_I_m	Dia_S_m	0.566	0.237	0.779	3.434	25	27	0.002	0.011
smD_I_m	AreaMito_I_m	0.901	0.792	0.954	10.380	25	27	0.000	0.000
smD_I_m	AreaMito_S_m	0.724	0.474	0.866	5.243	25	27	0.000	0.000
smD_I_m	AreaMitoTotal_I_m	0.287	-0.104	0.602	1.501	25	27	0.146	0.367
smD_I_m	AreaMitoTotal_S_m	0.298	-0.093	0.609	1.560	25	27	0.131	0.360
smD_I_m	Vol_I_m	0.615	0.307	0.807	3.901	25	27	0.001	0.004
smD_I_m	Vol_S_m	0.294	-0.097	0.606	1.538	25	27	0.137	0.363
smD_I_m	Sar_I_m	0.167	-0.227	0.515	0.849	25	27	0.404	0.598
smD_I_m	IB_I_m	0.183	-0.212	0.526	0.930	25	27	0.361	0.558
smD_I_m	AB_I_m	0.145	-0.249	0.497	0.732	25	27	0.471	0.664
smD_I_m	Mito_Vol%_Mus_Total	0.440	0.072	0.703	2.449	25	27	0.022	0.086
smD_I_m	Mito_Vol%_Mus_I	0.617	0.310	0.808	3.922	25	27	0.001	0.003
smD_I_m	Mito_Vol%_Mus_S	0.294	-0.097	0.606	1.538	25	27	0.137	0.363
smD_S_m	meD_I_m	0.784	0.575	0.897	6.311	25	27	0.000	0.000
smD_S_m	meD_S_m	0.897	0.785	0.953	10.173	25	27	0.000	0.000
smD_S_m	Dia_I_m	0.716	0.462	0.862	5.128	25	27	0.000	0.000
smD_S_m	Dia_S_m	0.836	0.669	0.923	7.627	25	27	0.000	0.000
smD_S_m	AreaMito_I_m	0.673	0.393	0.838	4.544	25	27	0.000	0.001
smD_S_m	AreaMito_S_m	0.956	0.905	0.980	16.293	25	27	0.000	0.000
smD_S_m	AreaMitoTotal_I_m	0.257	-0.136	0.580	1.331	25	27	0.195	0.426

Parameter 1	Parameter 2	r	CI_low	CI_high	t	df	N	p	p_{fdr}
smD_S_m	AreaMitoTotal_S_m	0.682	0.408	0.844	4.669	25	27	0.000	0.001
smD_S_m	Vol_I_m	0.508	0.158	0.744	2.947	25	27	0.007	0.032
smD_S_m	Vol_S_m	0.676	0.398	0.840	4.584	25	27	0.000	0.001
smD_S_m	Sar_I_m	0.134	-0.259	0.489	0.677	25	27	0.505	0.690
smD_S_m	IB_I_m	0.154	-0.240	0.505	0.781	25	27	0.442	0.633
smD_S_m	AB_I_m	0.189	-0.205	0.531	0.965	25	27	0.344	0.554
smD_S_m	Mito_Vol%_Mus_Total	0.711	0.453	0.859	5.051	25	27	0.000	0.000
smD_S_m	Mito_Vol%_Mus_I	0.504	0.153	0.742	2.916	25	27	0.007	0.034
smD_S_m	Mito_Vol%_Mus_S	0.676	0.398	0.840	4.584	25	27	0.000	0.001
meD_I_m	meD_S_m	0.695	0.429	0.851	4.838	25	27	0.000	0.000
meD_I_m	Dia_I_m	0.918	0.826	0.962	11.569	25	27	0.000	0.000
meD_I_m	Dia_S_m	0.587	0.266	0.791	3.624	25	27	0.001	0.007
meD_I_m	AreaMito_I_m	0.941	0.873	0.973	13.896	25	27	0.000	0.000
meD_I_m	AreaMito_S_m	0.768	0.548	0.889	5.992	25	27	0.000	0.000
meD_I_m	AreaMitoTotal_I_m	0.311	-0.078	0.618	1.637	25	27	0.114	0.321
meD_I_m	AreaMitoTotal_S_m	0.377	-0.003	0.662	2.036	25	27	0.052	0.184
meD_I_m	Vol_I_m	0.656	0.368	0.830	4.351	25	27	0.000	0.001
meD_I_m	Vol_S_m	0.368	-0.014	0.656	1.976	25	27	0.059	0.204
meD_I_m	Sar_I_m	0.327	-0.061	0.629	1.729	25	27	0.096	0.288
meD_I_m	IB_I_m	0.226	-0.169	0.558	1.158	25	27	0.258	0.492
meD_I_m	AB_I_m	0.360	-0.023	0.651	1.929	25	27	0.065	0.222
meD_I_m	Mito_Vol%_Mus_Total	0.496	0.143	0.737	2.858	25	27	0.008	0.038
meD_I_m	Mito_Vol%_Mus_I	0.653	0.363	0.828	4.312	25	27	0.000	0.001
meD_I_m	Mito_Vol%_Mus_S	0.368	-0.014	0.656	1.976	25	27	0.059	0.204
meD_S_m	Dia_I_m	0.630	0.328	0.815	4.052	25	27	0.000	0.003
meD_S_m	Dia_S_m	0.929	0.849	0.968	12.583	25	27	0.000	0.000
meD_S_m	AreaMito_I_m	0.559	0.227	0.775	3.372	25	27	0.002	0.012
meD_S_m	AreaMito_S_m	0.938	0.867	0.972	13.546	25	27	0.000	0.000
meD_S_m	AreaMitoTotal_I_m	0.203	-0.191	0.542	1.038	25	27	0.309	0.518
meD_S_m	AreaMitoTotal_S_m	0.694	0.427	0.850	4.823	25	27	0.000	0.000
meD_S_m	Vol_I_m	0.450	0.085	0.709	2.522	25	27	0.018	0.075
meD_S_m	Vol_S_m	0.687	0.416	0.846	4.729	25	27	0.000	0.001
meD_S_m	Sar_I_m	0.293	-0.098	0.605	1.530	25	27	0.139	0.363
meD_S_m	IB_I_m	0.100	-0.292	0.462	0.500	25	27	0.621	0.779
meD_S_m	AB_I_m	0.359	-0.024	0.650	1.924	25	27	0.066	0.222
meD_S_m	Mito_Vol%_Mus_Total	0.655	0.366	0.829	4.335	25	27	0.000	0.001

Parameter 1	Parameter 2	r	CI_low	CI_high	t	df	N	p	p_{fdr}
meD_S_m	Mito_Vol%_Mus_I	0.449	0.083	0.708	2.513	25	27	0.019	0.076
meD_S_m	Mito_Vol%_Mus_S	0.687	0.416	0.846	4.729	25	27	0.000	0.001
Dia_I_m	Dia_S_m	0.656	0.368	0.830	4.351	25	27	0.000	0.001
Dia_I_m	AreaMito_I_m	0.875	0.742	0.942	9.050	25	27	0.000	0.000
Dia_I_m	AreaMito_S_m	0.654	0.365	0.828	4.328	25	27	0.000	0.001
Dia_I_m	AreaMitoTotal_I_m	0.344	-0.041	0.641	1.834	25	27	0.079	0.246
Dia_I_m	AreaMitoTotal_S_m	0.271	-0.121	0.591	1.410	25	27	0.171	0.403
Dia_I_m	Vol_I_m	0.637	0.338	0.819	4.127	25	27	0.000	0.002
Dia_I_m	Vol_S_m	0.262	-0.131	0.584	1.358	25	27	0.187	0.420
Dia_I_m	Sar_I_m	0.248	-0.146	0.574	1.279	25	27	0.213	0.455
Dia_I_m	IB_I_m	0.124	-0.268	0.482	0.627	25	27	0.537	0.707
Dia_I_m	AB_I_m	0.277	-0.116	0.594	1.439	25	27	0.163	0.396
Dia_I_m	Mito_Vol%_Mus_Total	0.421	0.049	0.691	2.322	25	27	0.029	0.111
Dia_I_m	Mito_Vol%_Mus_I	0.634	0.335	0.817	4.102	25	27	0.000	0.002
Dia_I_m	Mito_Vol%_Mus_S	0.262	-0.131	0.584	1.358	25	27	0.187	0.420
Dia_S_m	AreaMito_I_m	0.450	0.085	0.709	2.520	25	27	0.018	0.075
Dia_S_m	AreaMito_S_m	0.844	0.683	0.927	7.874	25	27	0.000	0.000
Dia_S_m	AreaMitoTotal_I_m	0.199	-0.196	0.539	1.017	25	27	0.319	0.531
Dia_S_m	AreaMitoTotal_S_m	0.609	0.298	0.803	3.842	25	27	0.001	0.004
Dia_S_m	Vol_I_m	0.409	0.034	0.683	2.242	25	27	0.034	0.130
Dia_S_m	Vol_S_m	0.602	0.288	0.799	3.773	25	27	0.001	0.005
Dia_S_m	Sar_I_m	0.213	-0.182	0.549	1.090	25	27	0.286	0.502
Dia_S_m	IB_I_m	0.046	-0.340	0.419	0.233	25	27	0.818	0.903
Dia_S_m	AB_I_m	0.287	-0.104	0.601	1.498	25	27	0.147	0.367
Dia_S_m	Mito_Vol%_Mus_Total	0.590	0.271	0.793	3.656	25	27	0.001	0.006
Dia_S_m	Mito_Vol%_Mus_I	0.407	0.032	0.682	2.228	25	27	0.035	0.131
Dia_S_m	Mito_Vol%_Mus_S	0.602	0.288	0.799	3.773	25	27	0.001	0.005
AreaMito_I_m	AreaMito_S_m	0.640	0.343	0.820	4.164	25	27	0.000	0.002
AreaMito_I_m	AreaMitoTotal_I_m	0.179	-0.216	0.524	0.911	25	27	0.371	0.560
AreaMito_I_m	AreaMitoTotal_S_m	0.291	-0.100	0.604	1.523	25	27	0.140	0.365
AreaMito_I_m	Vol_I_m	0.626	0.323	0.813	4.017	25	27	0.000	0.003
AreaMito_I_m	Vol_S_m	0.287	-0.104	0.601	1.499	25	27	0.147	0.367
AreaMito_I_m	Sar_I_m	0.258	-0.136	0.581	1.334	25	27	0.194	0.426
AreaMito_I_m	IB_I_m	0.210	-0.185	0.546	1.072	25	27	0.294	0.514
AreaMito_I_m	AB_I_m	0.260	-0.133	0.583	1.348	25	27	0.190	0.420

Parameter 1	Parameter 2	r	CI_low	CI_high	t	df	N	p	p_{fdr}
AreaMito_I_m	Mito_Vol%_Mus_Total	0.425	0.054	0.693	2.349	25	27	0.027	0.105
AreaMito_I_m	Mito_Vol%_Mus_I	0.624	0.319	0.811	3.988	25	27	0.001	0.003
AreaMito_I_m	Mito_Vol%_Mus_S	0.287	-0.104	0.601	1.499	25	27	0.147	0.367
AreaMito_S_m	AreaMitoTotal_I_m	0.245	-0.148	0.572	1.266	25	27	0.217	0.459
AreaMito_S_m	AreaMitoTotal_S_m	0.733	0.489	0.871	5.388	25	27	0.000	0.000
AreaMito_S_m	Vol_I_m	0.509	0.160	0.745	2.954	25	27	0.007	0.032
AreaMito_S_m	Vol_S_m	0.724	0.474	0.866	5.240	25	27	0.000	0.000
AreaMito_S_m	Sar_I_m	0.215	-0.179	0.550	1.103	25	27	0.281	0.495
AreaMito_S_m	IB_I_m	0.204	-0.191	0.542	1.042	25	27	0.307	0.518
AreaMito_S_m	AB_I_m	0.297	-0.094	0.608	1.555	25	27	0.133	0.361
AreaMito_S_m	Mito_Vol%_Mus_Total	0.731	0.487	0.870	5.362	25	27	0.000	0.000
AreaMito_S_m	Mito_Vol%_Mus_I	0.502	0.151	0.741	2.904	25	27	0.008	0.034
AreaMito_S_m	Mito_Vol%_Mus_S	0.724	0.474	0.866	5.240	25	27	0.000	0.000
AreaMitoTotal_I_m	AreaMitoTotal_S_m	0.044	-0.342	0.417	0.221	25	27	0.827	0.904
AreaMitoTotal_I_m	Vol_I_m	0.381	0.001	0.665	2.062	25	27	0.050	0.178
AreaMitoTotal_I_m	Vol_S_m	0.038	-0.347	0.412	0.189	25	27	0.852	0.917
AreaMitoTotal_I_m	Sar_I_m	0.118	-0.274	0.477	0.597	25	27	0.556	0.717
AreaMitoTotal_I_m	IB_I_m	-0.064	-0.433	0.324	-0.321	25	27	0.751	0.867
AreaMitoTotal_I_m	AB_I_m	0.195	-0.200	0.535	0.993	25	27	0.330	0.542
AreaMitoTotal_I_m	Mito_Vol%_Mus_Total	0.207	-0.188	0.544	1.056	25	27	0.301	0.518
AreaMitoTotal_I_m	Mito_Vol%_Mus_I	0.378	-0.003	0.663	2.040	25	27	0.052	0.184
AreaMitoTotal_I_m	Mito_Vol%_Mus_S	0.038	-0.347	0.412	0.189	25	27	0.852	0.917
AreaMitoTotal_S_m	Vol_I_m	0.320	-0.068	0.624	1.690	25	27	0.104	0.306
AreaMitoTotal_S_m	Vol_S_m	0.999	0.998	1.000	129.692	25	27	0.000	0.000
AreaMitoTotal_S_m	Sar_I_m	0.139	-0.255	0.493	0.701	25	27	0.490	0.682
AreaMitoTotal_S_m	IB_I_m	0.062	-0.325	0.432	0.313	25	27	0.757	0.871
AreaMitoTotal_S_m	AB_I_m	0.234	-0.160	0.564	1.203	25	27	0.240	0.480
AreaMitoTotal_S_m	Mito_Vol%_Mus_Total	0.938	0.868	0.972	13.560	25	27	0.000	0.000

Parameter 1	Parameter 2	r	CI_low	CI_high	t	df	N	p	p_{fdr}
AreaMitoTotal_S_m	Mito_Vol%_Mus_I	0.312	-0.077	0.619	1.644	25	27	0.113	0.321
AreaMitoTotal_S_m	Mito_Vol%_Mus_S	0.999	0.998	1.000	129.692	25	27	0.000	0.000
Vol_I_m	Vol_S_m	0.314	-0.075	0.620	1.652	25	27	0.111	0.320
Vol_I_m	Sar_I_m	0.190	-0.204	0.532	0.970	25	27	0.341	0.554
Vol_I_m	IB_I_m	0.081	-0.308	0.448	0.409	25	27	0.686	0.828
Vol_I_m	AB_I_m	0.180	-0.214	0.524	0.916	25	27	0.368	0.560
Vol_I_m	Mito_Vol%_Mus_Total	0.550	0.215	0.769	3.295	25	27	0.003	0.014
Vol_I_m	Mito_Vol%_Mus_I	1.000	0.999	1.000	192.996	25	27	0.000	0.000
Vol_I_m	Mito_Vol%_Mus_S	0.314	-0.075	0.620	1.652	25	27	0.111	0.320
Vol_S_m	Sar_I_m	0.127	-0.266	0.483	0.638	25	27	0.530	0.704
Vol_S_m	IB_I_m	0.050	-0.336	0.422	0.252	25	27	0.803	0.895
Vol_S_m	AB_I_m	0.222	-0.173	0.555	1.137	25	27	0.266	0.494
Vol_S_m	Mito_Vol%_Mus_Total	0.937	0.865	0.971	13.441	25	27	0.000	0.000
Vol_S_m	Mito_Vol%_Mus_I	0.306	-0.084	0.614	1.606	25	27	0.121	0.334
Vol_S_m	Mito_Vol%_Mus_S	1.000	1.000	1.000	Inf	25	27	0.000	0.000
Sar_I_m	IB_I_m	0.557	0.224	0.773	3.353	25	27	0.003	0.013
Sar_I_m	AB_I_m	0.956	0.905	0.980	16.342	25	27	0.000	0.000
Sar_I_m	Mito_Vol%_Mus_Total	0.091	-0.299	0.455	0.456	25	27	0.652	0.806
Sar_I_m	Mito_Vol%_Mus_I	0.195	-0.200	0.535	0.995	25	27	0.329	0.542
Sar_I_m	Mito_Vol%_Mus_S	0.127	-0.266	0.483	0.638	25	27	0.530	0.704
IB_I_m	AB_I_m	0.438	0.070	0.701	2.437	25	27	0.022	0.088
IB_I_m	Mito_Vol%_Mus_Total	-0.016	-0.393	0.367	-0.078	25	27	0.938	0.963
IB_I_m	Mito_Vol%_Mus_I	0.078	-0.311	0.445	0.394	25	27	0.697	0.832
IB_I_m	Mito_Vol%_Mus_S	0.050	-0.336	0.422	0.252	25	27	0.803	0.895
AB_I_m	Mito_Vol%_Mus_Total	0.185	-0.210	0.528	0.939	25	27	0.357	0.554
AB_I_m	Mito_Vol%_Mus_I	0.180	-0.215	0.524	0.915	25	27	0.369	0.560
AB_I_m	Mito_Vol%_Mus_S	0.222	-0.173	0.555	1.137	25	27	0.266	0.494
Mito_Vol%_Mus_Total	Mito_Vol%_Mus_I	0.543	0.205	0.765	3.233	25	27	0.003	0.016
Mito_Vol%_Mus_Total	Mito_Vol%_Mus_S	0.937	0.865	0.971	13.441	25	27	0.000	0.000
Mito_Vol%_Mus_I	Mito_Vol%_Mus_S	0.306	-0.084	0.614	1.606	25	27	0.121	0.334

Supplementary Table S2

Group differences (bootstrapped results).

	$M (SD)_{CFS}$	$M (SD)_{PCS}$	T_{Diff}	T_y	p_y	δ_t	95%-CI $_{\delta t}$
Shape $_{intermyofibrillar}$	1.40 (0.30)	1.42 (0.27)	-0.02	-0.12	0.887	-0.05	[-0.74, 0.95]
Shape $_{subsarcolemmal}$	1.50 (0.24)	1.65 (0.31)	-0.16	-1.05	0.237	-0.44	[-1.2, 0.34]
Cristae-Score $_{intermyofibrillar}$	1.56 (0.69)	2.70 (0.96)	-1.33	-3.61	**0.001**	-1.52	[-2.89, -0.55]
Cristae-Score $_{subsarcolemmal}$	1.69 (0.82)	2.97 (0.82)	-1.39	-4.08	**0.001**	-1.67	[-3.02, -0.77]
minimal Feret´s Diameter $_{intermyofibrillar}$	0.23 (0.04)	0.25 (0.05)	-0.01	-0.37	0.682	-0.15	[-0.84, 0.71]
minimal Feret´s Diameter $_{subsarcolemmal}$	0.25 (0.04)	0.27 (0.05)	-0.03	-1.24	0.166	-0.51	[-1.29, 0.22]
Feret´s Diameter $_{intermyofibrillar}$	0.41 (0.09)	0.45 (0.09)	-0.02	-0.55	0.533	-0.22	[-0.98, 0.48]
Feret´s Diameter $_{subsarcolemmal}$	0.43 (0.07)	0.50 (0.08)	-0.07	-2.02	**0.037**	-0.83	[-1.97, -0.17]
Perimeter $_{intermyofibrillar}$	1.03 (0.19)	1.15 (0.24)	-0.09	-0.93	0.291	-0.38	[-1.19, 0.5]
Perimeter $_{subsarcolemmal}$	1.11 (0.17)	1.27 (0.20)	-0.21	-2.54	**0.018**	-1.06	[-2.53, -0.18]
Mean Area $_{intermyofibrillar}$	0.09 (0.04)	0.09 (0.04)	-0.01	-0.45	0.605	-0.18	[-0.9, 0.4]
Mean Area $_{subsarcolemmal}$	0.090 (0.031)	0.107 (0.031)	-0.02	-1.49	0.106	-0.61	[-1.83, 0.15]
Total Area $_{intermyofibrillar}$	0.66 (0.60)	0.62 (0.25)	-0.08	-0.58	0.511	-0.24	[-1.14, 0.39]
Total Area $_{subsarcolemmal}$	0.98 (0.55)	1.47 (0.66)	-0.5	-2.45	**0.016**	-1.03	[-1.79, -0.07]
Volume fraction $_{intermyofibrillar}$	0.053 (0.019)	0.056 (0.017)	0	-0.68	0.442	-0.28	[-0.96, 0.51]

Volume fraction $_{subsarcolemmal}$	0.08 (0.04)	0.13 (0.07)	-0.05	-1.88	0.076	-0.8	[-1.74, -0.14]
Sarcomere length (µm)	1.81 (0.27)	1.83 (0.29)	0.02	0.17	0.851	0.07	[-0.6, 0.82]
Length I-band (µm)	0.46 (0.07)	0.43 (0.09)	0.03	1.62	0.068	0.66	[-0.1, 1.37]
Length A-band (µm)	1.35 (0.22)	1.39 (0.19)	-0.04	-0.4	0.643	-0.16	[-0.99, 0.58]
Mito_Vol%_Mus_Total	0.065 (0.026)	0.087 (0.026)	-0.03	-2.18	**0.024**	-0.9	[-2.03, -0.2]
Mito_Vol%_Mus_I$_{ntermyofibrillar}$	0.053 (0.019)	0.056 (0.017)	-0.00	-0.65	0.473	-0.27	[-0.98, 0.43]
Mito_Vol%_Mus_S$_{ubsarcolemmal}$	0.08 (0.04)	0.12 (0.05)	-0.04	-2.43	**0.017**	-1.02	[-1.88, -0.19]

Notes. Mean (SD), Bootstrapped p-values (WRS2::yuenbt), significant results ($p \leq 0.05$) are highlighted in bold Effect size (**δ_t**): robust version of Cohen's d (Cohen 1988) proposed by Algina, Keselman, and Penfield (2005); $N_{CFS} = 15$, $N_{PCS} = 15$
